# Carotid Endarterectomy in the Era of Stenting and Optimal Medical Therapy

**DOI:** 10.7759/cureus.102683

**Published:** 2026-01-31

**Authors:** Ítalo Eugênio Souza Gadelha de Abreu, Matheus Eugênio de Sousa Lima, João Brainer Clares de Andrade

**Affiliations:** 1 Vascular Surgery, Hospital Geral de Fortaleza, Fortaleza, BRA; 2 Graduate Program in Health Sciences, Santa Casa de São Paulo, São Paulo, BRA; 3 Family and Community Medicine/Psychiatry, Messejana Psychiatric Hospital, Fortaleza, BRA; 4 Neurology, Federal University of São Paulo, São Paulo, BRA

**Keywords:** atherosclerotic, carotid, endarterectomy, image, stenosis

## Abstract

Cardiovascular events, including stroke and acute myocardial infarction (AMI), remain the leading causes of death worldwide. This study aimed to review the scientific literature evaluating carotid endarterectomy as a first-line intervention for carotid artery disease. A literature review was conducted, including 40 articles published between 1977 and 2025, retrieved from Scientific Electronic Library Online (SciELO), Google Scholar, and MEDLINE (PubMed).

Patients with carotid atherosclerotic disease should undergo individualized stroke risk assessment using validated imaging modalities to guide treatment selection. This review aimed to evaluate the current evidence regarding carotid endarterectomy (CEA) within the contemporary management of carotid artery stenosis.

A structured review of 40 studies published between 1977 and 2025 was conducted.. The analyzed literature demonstrates that CEA provides a clear benefit in patients with symptomatic carotid stenosis ≥70%, and selected patients with 50-69% stenosis, when performed in experienced centers with low perioperative risk, in accordance with current European Society for Vascular Surgery (ESVS) guidelines.

In contrast, for asymptomatic carotid stenosis, recent evidence from the Carotid Revascularization Endarterectomy versus Stenting Trial-2 (CREST-2) era highlights the critical role of optimized medical therapy, with revascularization reserved for carefully selected high-risk patients. In this population, CEA should be considered only when the procedural risk is low and the life expectancy is sufficient to derive long-term benefit.

In conclusion, CEA remains an effective intervention for stroke prevention in appropriately selected patients, particularly those with symptomatic disease, while contemporary management increasingly emphasizes individualized decision-making and aggressive medical therapy, especially in asymptomatic patients.

## Introduction and background

Cardiovascular and cerebrovascular diseases remain the leading causes of morbidity and mortality worldwide, largely driven by modifiable risk factors such as dyslipidemia and hypertension. Contemporary carotid revascularization strategies are therefore guided by current epidemiology and evidence-based risk assessment rather than historical observations. Atherosclerosis has been observed across historical populations; however, contemporary carotid revascularization strategies are guided by current epidemiology and modern evidence-based risk assessment [1-3].

Risk stratification tools such as the Framingham Risk Score aid population-level assessment but are limited in predicting individual benefit, whereas lifestyle measures, including the Mediterranean diet, have been shown to slow atherosclerotic progression [4,5].

Advanced imaging is essential for identifying high-risk cerebrovascular plaque features. Carotid ultrasound has demonstrated an association between plaque ulceration and ischemic infarction [6-8], while more sophisticated techniques (such as computed tomography angiography, magnetic resonance imaging (MRI), and vessel-wall imaging) have contributed to safer therapeutic strategies, including carotid endarterectomy (CEA) [9].

The carotid arteries are essential for cerebral perfusion and are closely involved in the pathophysiology of ischemic stroke. In clinical practice, the anatomic characteristics of the carotid bifurcation represent key determinants of treatment selection, influencing the feasibility and risk profiles of CEA versus carotid artery stenting (CAS) [10]. Features such as a high carotid bifurcation, prior neck surgery or radiation, tracheostomy, or hostile neck anatomy may favor an endovascular approach, whereas standard anatomy is generally more appropriate for CEA. Contraindications and exclusion criteria for CAS, including contrast allergy not amenable to premedication, type III aortic arch anatomy, severe tortuosity or angulation of the aortic arch or carotid vessels, proximal or ostial common carotid disease, innominate artery stenosis, and distal intracranial stenosis exceeding the target lesion, are consistent with the 2023 European Society for Vascular Surgery (ESVS) clinical practice guidelines, as these factors are associated with increased procedural risk and reduced technical success [10].

CEA treats carotid artery stenosis by surgically removing atherosclerotic plaque to restore luminal patency and reduce the risk of ischemic stroke, and it remains the standard intervention for patients with significant stenosis when clinical or anatomic features favor surgery. Carotid artery stenting CAS is an endovascular alternative that uses percutaneous access to deploy a stent, often with balloon angioplasty, to maintain vessel diameter and limit embolization, and is primarily reserved for selected patients with increased surgical risk or anatomy less suitable for CEA [10,11].

In the contemporary management of carotid disease, the central question is how to define the clinical and anatomical scenarios in which carotid endarterectomy offers superior outcomes compared with carotid artery stenting [11].

## Review

Methodology

This review was structured using the PICO framework. The Population (P) consisted of adult patients (≥18 years) undergoing evaluation or treatment for carotid artery disease. The Interventions (I) included carotid endarterectomy, carotid artery stenting/angioplasty, and best medical therapy (BMT). The Comparator (C) was the standard surgical endarterectomy technique. The Outcomes (O) focused on procedural complications and identification of the most appropriate treatment strategy for carotid stenosis.

A comprehensive search was conducted across PubMed, SciELO, and Google Scholar covering publications from 1977 to 2025, supplemented by manual reference screening from relevant studies. The search strategy incorporated the following keywords and their combinations: “carotid stenosis,” “atherosclerotic stenosis,” “carotid endarterectomy,” “carotid stenting,” “endarterectomy,” “carotid imaging,” “Doppler ultrasound,” and “atherosclerotic plaque.” Boolean connectors (AND/OR) were applied to optimize the sensitivity and specificity of retrieved records.

**Figure 1:**
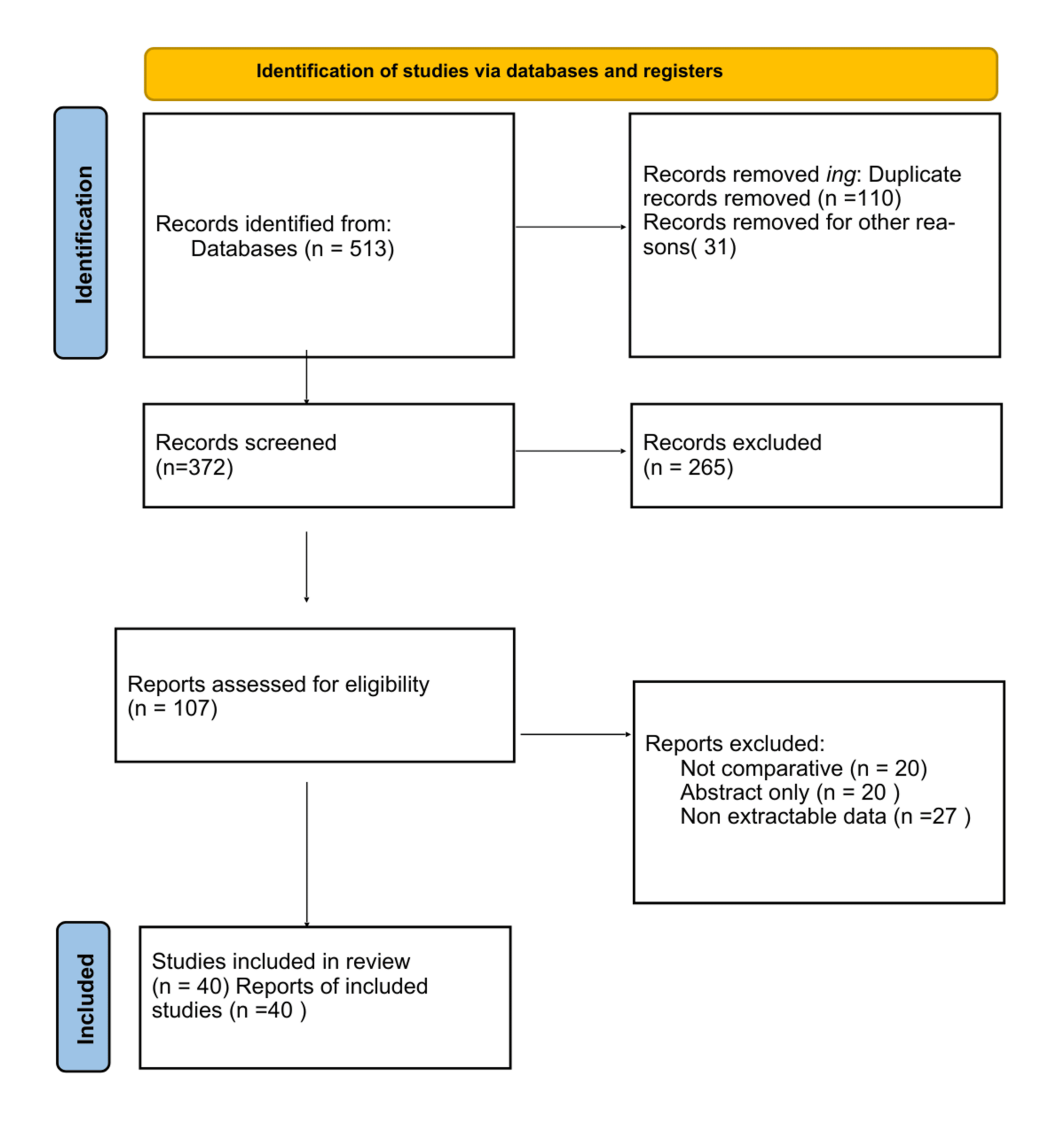
PRISMA flow diagram of study selection. PRISMA: Preferred Reporting Items for Systematic Reviews and Meta-Analyses.

The literature search identified 513 records (Figure 1). After removal of 110 duplicates and 31 clearly non-eligible entries, 372 records underwent title and abstract screening, of which 265 were excluded. A total of 107 full-text articles were assessed for eligibility, and 67 were excluded due to non-comparative design (n = 20), abstract-only publication (n = 20), or insufficient or non-extractable data (n = 27). Ultimately, 40 studies met the predefined criteria and were included in this narrative review, comprising randomized controlled trials, cohort studies, observational studies, systematic reviews, guidelines, and consensus statements relevant to the evaluation and management of carotid artery disease.

This study was conducted as a narrative literature review, identification and selection guided by Preferred Reporting Items for Systematic Reviews and Meta-Analyses (PRISMA) 2020 recommendations to enhance transparency (Figure 1, Table 1) [12,13]. As this review did not follow a systematic or meta-analytic methodology, no formal risk of bias assessment was performed; however, key methodological characteristics of the included literature were considered to contextualize the strength of the evidence.

**Table 1:** Summary of included studies and levels of evidence. ACAS: Asymptomatic Carotid Atherosclerosis Study; ACST: Asymptomatic Carotid Surgery Trial; BP: blood pressure; CAS: carotid artery stenting; CEA: carotid endarterectomy; CREST-2: Carotid Revascularization Endarterectomy versus Stenting Trial–2; ECST: European Carotid Surgery Trial; ESVS: European Society for Vascular Surgery; FMD: fibromuscular dysplasia; ICA: internal carotid artery; IPD: individual participant data; JACC: Journal of the American College of Cardiology; MRI: magnetic resonance imaging; OCEBM: Oxford Centre for Evidence-Based Medicine; PREDIMED: Prevención con Dieta Mediterránea; RCT: randomized controlled trial; SBC: Sociedade Brasileira de Cardiologia (Brazilian Society of Cardiology); TCAR: transcarotid artery revascularization. * OCEBM level of evidence (2011) [13].

Year	Authors	Study topic	Study design	OCEBM level of evidence (2011)*	Key focus	Citation number
2019	Mazzaccaro et al.	Assessment of long-term survival and stroke after CEA and CAS in octogenarians	Cohort study	2a	Long-term outcomes after CEA vs. CAS	[1]
2011	Allam et al.	Atherosclerosis in Ancient Egyptian mummies	Observational imaging study	2b	Plaque detection in ancient remains	[2]
2010	Naqvi et al.	High prevalence of carotid atherosclerosis in low Framingham score	Cross-sectional study	2b	Screening for subclinical disease	[3]
2017	Amor et al.	Prediction of cardiovascular disease in the PREDIMED cohort	Prospective cohort	2a	Diet and cardiovascular risk prediction	[4]
2014	Doménech et al.	Mediterranean diet reduces blood pressure, glucose, and lipids	Randomized controlled trial	1b	Dietary intervention outcomes	[5]
2017	Howard et al.	CREST-2 trial protocol	RCT protocol	2b	Trial design comparing CEA vs. CAS	[6]
2020	Yilmaz et al.	Diagnostic approach for stroke etiology in Takayasu arteritis	Case series	4	Diagnostic algorithm	[7]
2006	Takaya et al.	Carotid plaque characteristics and stroke risk	Prospective cohort	2a	MRI plaque predictors of stroke	[8]
2019	Gornik et al.	International consensus on fibromuscular dysplasia	Consensus guideline	1a	FMD management recommendations	[9]
2023	Naylor et al.	ESVS 2023 guidelines on carotid and vertebral artery disease	Guideline	1a	Management recommendations	[10]
2000	Chaves	Stroke: definition and risk factors	Narrative review	5	Stroke concepts and risk factors	[11]
2005	Giannoukas et al.	Management of near-total internal carotid artery occlusion	Review	2a	Near-occlusion treatment strategies	[14]
2017	Neves	Long-term outcomes in carotid string sign	Case series/thesis	4	ICA string sign outcomes	[15]
2019	Santos et al.	Brazilian Society of Cardiology ultrasound statement	Guideline	1a	Vascular ultrasound recommendations	[16]
1983	Reilly et al.	Carotid plaque histology via real-time ultrasonography	Observational study	2b	Ultrasound–histology correlation	[17]
2015	Freire et al.	Quantification of carotid and vertebral disease by ultrasound	Guideline	1a	Ultrasound quantification	[18]
2002	Lal et al.	Pixel distribution analysis predicts plaque histology	Observational imaging study	2b	Plaque characterization	[19]
2009	Oates et al.	Joint recommendations for carotid ultrasound	Guideline	1a	Ultrasound protocols	[20]
1999	Ferguson et al.	NASCET: surgical results in 1415 patients	Randomized controlled trial	1b	CEA outcomes in symptomatic stenosis	[21]
1991	Warlow et al.	MRC European Carotid Surgery Trial	Randomized controlled trial	1b	Surgery vs. medical therapy	[22]
2003	Rothwell et al.	Reanalysis of ECST final results	RCT reanalysis	1b	Prognostic refinement	[23]
2007	Ristow	The back-and-forth in carotid disease treatment	Narrative review	5	Historical perspective	[24]
2007	Bonamigo & Lucas	Critical analysis of carotid surgery indications	Narrative review	5	Surgical indications	[25]
2023	Leung et al.	Safety of CEA by age: IPD meta-analysis	Meta-analysis	1a	Age-related outcomes	[26]
2007	Zacharias et al.	Carotid stenting in subtotal ICA occlusion	Case series	4	CAS feasibility	[27]
2021	Halliday et al.	ACST-2 trial	Randomized controlled trial	1b	CAS vs. CEA in asymptomatic patients	[28]
1995	Walker et al.	ACAS trial	Randomized controlled trial	1b	CEA in asymptomatic stenosis	[29]
1993	Hobson et al.	Veterans Affairs Cooperative Study	Randomized controlled trial	1b	Efficacy of CEA	[30]
2005	Fox et al.	Carotid artery near occlusion	Cohort study	2a	Prognosis and management	[31]
2026	Brott et al.	Medical management vs. revascularization	Randomized controlled trial	1b	Modern BMT vs. intervention	[32]
2008	Curtis & Johansen	Techniques in carotid surgery	Narrative review	5	Surgical techniques	[33]
1977	Cossman et al.	ICA back pressure technique	Case series	4	Intraoperative technique	[34]
1989	Brott et al.	Stroke neurological assessment scale	Randomized controlled trial	1b	CEA efficacy	[35]
1988	van Swieten et al.	Interobserver agreement in stroke disability	Observational study	2b	Outcome assessment	[36]
2023	Vuurberg et al.	Monitoring during CEA: systematic review	Systematic review	1a	Perioperative monitoring	[37]
2008	Gurm et al.	Long-term CAS vs. CEA in high-risk patients	Randomized controlled trial	1b	High-risk revascularization	[38]
2021	Cui et al.	Timing effects after TCAR	Retrospective cohort	2b	TCAR outcomes	[39]
2024	Suh et al.	TCAR-related dissections (FDA database)	Observational registry study	2b	TCAR complications	[40]

Eligibility Criteria

Studies were included if they evaluated carotid endarterectomy, carotid artery stenting, or best medical therapy in adult patients with carotid artery disease and reported clinical outcomes related to stroke or perioperative events. English-language articles published between 1977 and 2025 were eligible. Abstract-only publications, case reports, editorial articles, and non-clinical or experimental studies were excluded.

Etiology

Atherosclerosis is the primary driver of TIA and stroke from extracranial artery disease. The formation of atherosclerotic plaque and its progressive encroachment on the vessel lumen are considered part of a degenerative process affecting the arterial wall. The extracranial carotid segments most frequently involved include the carotid bifurcation, the common carotid artery, the subclavian artery, and the vertebral arteries.

Takaya et al. reported that cerebral ischemia may be associated not only with plaque morphology but also with intraplaque processes, such as microembolization arising from intraplaque hemorrhage [8,14].

Among the rare causes of cerebrovascular disease in young women presenting with symptoms such as limb claudication, cervical pain, and differences in upper-limb systolic blood pressure, Takayasu arteritis should be considered as a potential diagnosis [8,15]. Fibromuscular dysplasia is another uncommon non-atherosclerotic etiology of stroke, affecting the carotid, vertebral, and intracranial arteries, and may lead to aneurysms and dissections, particularly in female patients [4,15].

Pathophysiology

Stroke is characterized by neurological dysfunction resulting from injury to the central nervous system and is classified as either ischemic (caused by obstruction of arterial blood flow due to thrombotic or embolic mechanisms) or hemorrhagic, which includes intraparenchymal and subarachnoid hemorrhage resulting from rupture of intracranial vessels. Ischemic strokes are further categorized into lacunar, embolic, and atherosclerotic subtypes [11,12,16].

Cryptogenic infarction refers to a stroke in which routine neuroimaging and diagnostic evaluation fail to identify an underlying cause, a scenario that may account for 30% or more of all ischemic stroke cases.

Giannoukas et al. highlighted that the primary risk factors included in the Framingham score are smoking, dyslipidemia, hypertension, and diabetes mellitus [14]. Even in patients with a low Framingham risk score, the authors recommend carotid Doppler ultrasound assessment. If no carotid plaques are detected, repeat evaluation is advised every three to five years; if plaques are present, annual follow-up is recommended [14].

Additional factors that may contribute to transient ischemic attack and stroke include variables such as the adequacy of collateral circulation, embolus size, histopathological characteristics of the embolic material, final clot location, degree of reduction in cerebral blood flow, and duration of ischemia. These factors help explain the wide variability in clinical presentation and outcomes among affected patients [14,15].

Imaging Evaluation Methods

Carotid Doppler ultrasound is widely used in the assessment of cardiovascular risk because it allows measurement of intima-media thickness (IMT), evaluation of plaque morphology, and quantification of the degree of stenosis (parameters associated with cerebrovascular event risk) [14,15].

Current imaging modalities used in the evaluation of carotid artery disease include Doppler and pixel-based ultrasonography, magnetic resonance imaging (MRI), and computed tomographic angiography. Intima media thickness (IMT) measurement plays a well-established role in cardiovascular risk stratification and in the assessment of atherosclerotic plaque characteristics [16]. Nevertheless, in the setting of critical carotid stenosis, an IMT value <1.5 mm should not be considered a reliable determinant of surgical risk [16,17].

Evaluation of stenosis severity requires integration of two-dimensional B-mode imaging with color Doppler assessment. Stenosis exceeding 50% warrants more detailed analysis, including plaque location, extension, echogenicity, and echotexture [16]. In color Doppler ultrasound, stenosis quantification is based on spectral Doppler flow velocities and changes in blood velocity attributable to proximal or distal hemodynamic effects [14-16].

Freire et al. recommend that Doppler velocity measurements be performed in a straight arterial segment free of plaque [18]. For analysis of the internal carotid artery (ICA), measurements should be taken in the proximal and mid segments, as atherosclerotic plaques commonly occur within the first 2 cm of the ICA. Measurements of the common carotid artery (CCA) should be performed approximately 2 cm proximal to the bifurcation. The transducer should remain parallel to the vessel, with an insonation angle below 60 degrees.

Regarding the “string sign,” Freire et al. note that subocclusion may present with high, low, or undetectable flow velocities, making diagnostic interpretation difficult [18]. Differentiating subocclusion from complete occlusion is essential, as this distinction significantly influences patient management and therapeutic decision-making [19].

In pixel-based B-mode ultrasound imaging of atherosclerotic plaques, red areas indicate intraplaque hemorrhage, yellow represents lipid components, green corresponds to fibromuscular tissue, and blue denotes gray-scale median (GSM) values of 211-255 [19].

In their evaluation, Oates et al. contrasted the North American Symptomatic Carotid Endarterectomy Trial (NASCET) and European Carotid Surgery Trial (ECST) methods for quantifying carotid stenosis, noting that each relies on a distinct geometric calculation [20-22].

NASCET calculates stenosis as:

${NASCET (\%)} = \left( \frac{D_{\text{distal ICA}} - D_{\mathrm{stenosis}}}{D_{\text{distal ICA}}} \right) \times 100$

ECST, in contrast, uses the estimated original vessel diameter:

${ECST (\%)} = \left( \frac{D_{\mathrm{original}} - D_{\mathrm{stenosis}}}{D_{\mathrm{original}}} \right) \times 100$

The authors further cite Rothwell et al. [23], who demonstrated that the ECST method systematically yields higher estimates of severe stenosis, following the approximate conversion formula (Table 2).

**Table 2:** NASCET and ECST methods for calculating carotid stenosis. Comparison of diagnostic definitions, formulas, and key characteristics of the NASCET and ECST stenosis grading methods. NASCET: North American Symptomatic Carotid Endarterectomy Trial; ECST: European Carotid Surgery Trial; ICA: Internal Carotid Artery.

Method	Definition	Formula for stenosis (%)	Notes
NASCET	Stenosis is calculated using the residual lumen at the point of maximal narrowing, compared with the normal distal ICA diameter	See NASCET formula below	Uses the distal ICA as reference; tends to give lower stenosis percentages than ECST
ECST	Stenosis is calculated using the estimated original normal diameter at the site of disease	See ECST formula below	May overestimate stenosis severity, especially in high-grade lesions
Approximate conversion	Provides approximate equivalence between NASCET and ECST measurements	See conversion formulas below	Useful for comparing studies using different criteria

Formulas used in Table 2:

$NASCET~(\%) = \left( \frac{D_{\text{distal ICA}} - D_{\mathrm{stenosis}}}{D_{\text{distal ICA}}} \right) \times 100​$

$ECST~(\%) = \left( \frac{D_{\mathrm{original}} - D_{\mathrm{stenosis}}}{D_{\mathrm{original}}} \right) \times 100​$

Approximate conversion:

$ECST (\%) \approx 0.6 \times NASCET (\%) + 40$ 

$NASCET (\%) \approx \frac{ECST (\%) - 40}{0.6}$

Controversy remains regarding the clinical benefit of performing carotid endarterectomy (CEA) within the first 48 hours after symptom onset. In the original ECST analysis, Rothwell et al. reported a stenosis of 85% on selective arteriography [23].

In a cohort of 1,216 patients with ICA stenosis >70%, Fox et al. emphasized the importance of diagnosing subocclusion using both NASCET and ECST criteria [21-23,31]. Key parameters include delayed contrast filling relative to external carotid branches and intracranial collaterals, reduced ICA diameter compared with the contralateral side, contrast dilution on the ipsilateral side, and comparison with the ipsilateral external carotid diameter.

Indications and Treatments

Regarding surgical indications, Ristow [24], in agreement with Bonamigo & Lucas [25], current guidelines recommend CEA only in carefully selected asymptomatic patients with ≥70% stenosis, sufficient life expectancy, and an expected perioperative stroke/death risk <3%; contemporary evidence from ESVS 2023 and Carotid Revascularization Endarterectomy versus Stenting Trial-2 (CREST-2) underscores that, with optimized medical therapy, the absolute benefit is modest and requires strict patient selection [6,10].

For symptomatic carotid disease, CEA provides a clear benefit in 70-99% stenosis and a more modest, sex-dependent benefit in 50-69% stenosis, with maximal benefit when performed within 14 days of symptom onset [21,24-26].

Leung et al. reported that approximately 10-15% of all strokes occur in the context of symptomatic carotid stenosis (50-99%), with symptomatic status defined by the presence of neurologic events within the preceding six months; patients without recent events are classified as asymptomatic [26,27].

For symptomatic patients, interventions performed within 14 days of symptom onset provide the greatest reduction in adverse events. Recommended medical management includes smoking cessation, physical activity, antiplatelet therapy, blood pressure control (including beta-blockers), glycemic management in diabetes, and continuous statin therapy.

The timing of intervention influences absolute risk reduction (ARR). Delayed surgery reduces the benefit: an ARR of 3.3% with a number needed to treat (NNT) of 30 is observed when CEA is performed within two to four weeks, whereas procedures performed between four and 12 weeks require a higher NNT (>30) to achieve a similar benefit [25,26].

Regarding CEA in octogenarians, evidence indicates that long-term risks of stroke and myocardial infarction are comparable to those in younger patients. Previous trials have shown that the risk of recurrent stroke increases substantially with age when patients are treated exclusively with best medical therapy [25-27].

Carotid Endarterectomy

Carotid endarterectomy (CEA) has been performed for more than half a century, providing a substantial body of evidence on its effectiveness and safety in long-term horizons. The major criteria supporting carotid endarterectomy were established through pivotal randomized trials.

Earlier randomized trials reported higher perioperative stroke and death rates, including 5.5% in NASCET, 7.5% in ECST, and 1.1% in the Veterans Affairs study; in asymptomatic populations, perioperative event rates of 2.3% and 3.1% were reported in Asymptomatic Carotid Atherosclerosis Study (ACAS) and Asymptomatic Carotid Surgery Trial (ACST), respectively, as summarized in the 2023 European Society for Vascular Surgery (ESVS) clinical practice guidelines [10,21,23,28-30]. Contemporary guidelines emphasize that the benefit of revascularization in asymptomatic carotid stenosis is modest in the context of optimized medical therapy and is contingent on maintaining a perioperative stroke and death risk below 3% [25,28-31].

Recent data from CREST-2 further support this paradigm. At four years, primary endpoint rates were approximately 6.0% with best medical therapy alone versus 2.8% with carotid artery stenting (relative risk = 3.2; number needed to treat = 31). Early peri-procedural events within 44 days occurred in approximately 1.3% of stented patients and were not observed in the medical therapy group. Similarly, four-year event rates were approximately 5.3% with best medical therapy versus 3.7% with carotid endarterectomy, while early events occurred in approximately 1.5% and 0.5% of patients, respectively. These findings suggest that although revascularization may reduce long-term ipsilateral stroke risk in selected asymptomatic patients, early procedural risk and the low annual stroke rates achieved with contemporary medical therapy support careful patient selection [31-33].

Differences in angiographic definitions of stenosis severity between NASCET and ECST led pooled analyses to demonstrate that carotid endarterectomy provides the greatest benefit in symptomatic patients with high-grade stenosis (70-99%), with an estimated number needed to treat of approximately six to prevent one stroke [21,22]. Additional populations appear to benefit disproportionately, including men, elderly patients over 75 years, those with contralateral occlusion, hemispheric neurological symptoms, or complex plaque features. Although concerns have been raised regarding how well these trial-level results translate to routine clinical practice, large real-world datasets (such as those derived from Medicare populations) have demonstrated similarly low complication rates, particularly when the procedure is performed by high-volume surgeons. The Medicare database between 1985 and 1996 revealed perioperative stroke and death rates as low as 1.6% and highlighted the strong influence of surgeon experience-rates were 1.9% for operators performing more than 50 procedures annually versus 2.5% for those with lower surgical volumes. Together, these data form the foundation for modern CEA indications and continue to guide decision-making when balancing surgical versus endovascular strategies, particularly in patients at standard operative risk [23-25,28,30,31,33].

Surgical Technique 

Carotid endarterectomy (CEA) is initiated by making an incision parallel to the medial edge of the sternocleidomastoid muscle and opening the platysma, providing adequate exposure of the carotid bifurcation for vascular dissection and control. The common, external, and internal carotid arteries are exposed, and vessel loops or cardiac tapes are applied for proximal and distal control. Meticulous identification and preservation of the vagus and hypoglossal nerves are essential. The thyrolinguofacial venous trunk is ligated when necessary, after which the common, external, and internal carotid arteries are sequentially clamped. 

**Figure 2:**
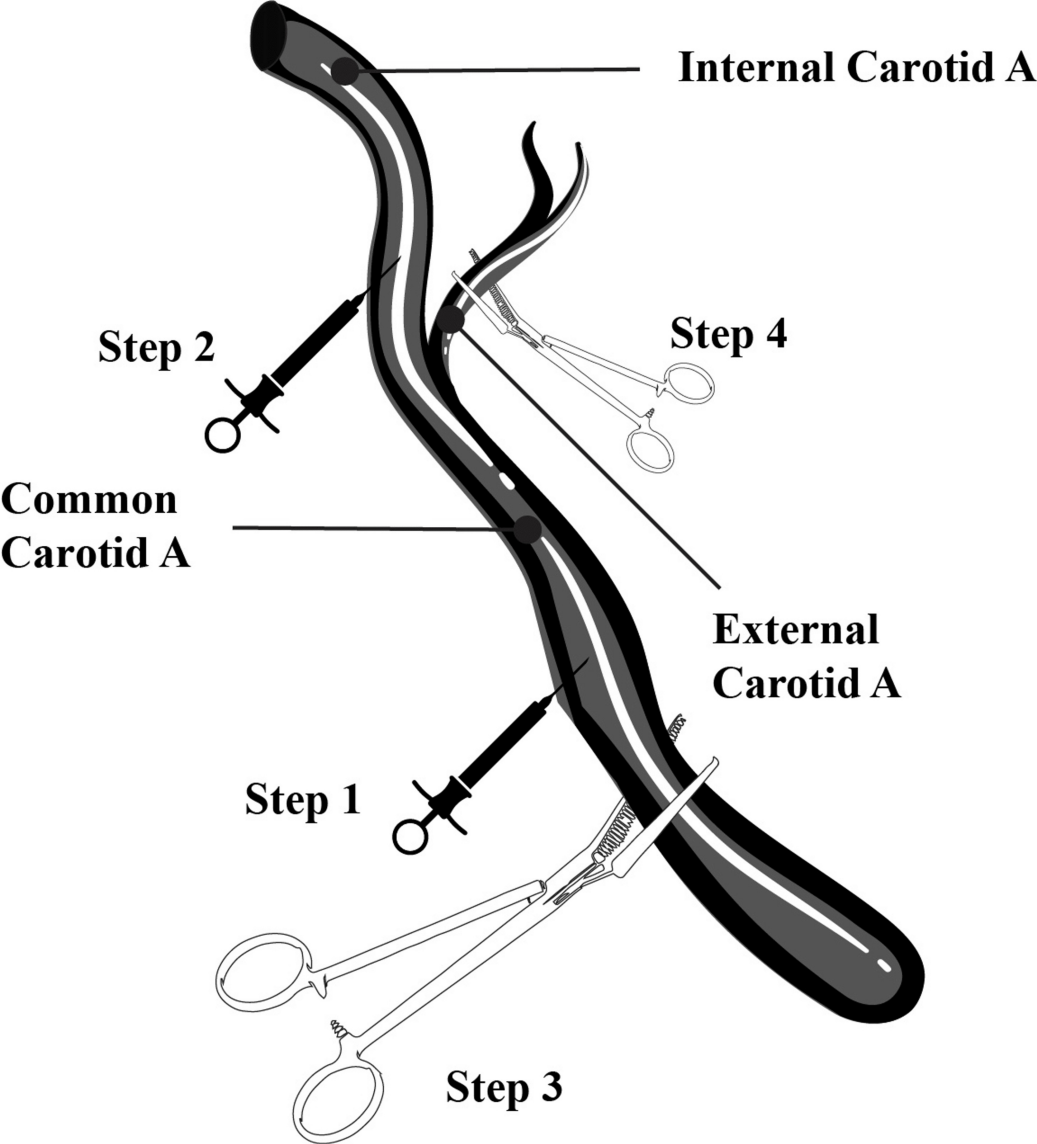
Conventional "two clamp — two needle" technique. Step 1: Needle pressure catheter inserted into the common carotid artery to measure pressure. Step 2: Needle pressure catheter inserted into the internal carotid artery above the lesion to assess the pressure gradient. Step 3/4: Common and external carotid arteries are cross-clamped to obtain the internal carotid stump pressure. Figure attribution: The illustration was originally designed and produced by the author.

Before arterial clamping, intravenous administration of 5,000 IU of sodium heparin is recommended. During the procedure, systemic blood pressure should be maintained approximately 20% above the patient’s baseline to ensure adequate collateral cerebral perfusion. Local anesthetic infiltration of the carotid body with lidocaine and bupivacaine may enhance hemodynamic stability (Figure 2).

The incision extends from the common into the internal carotid artery, requiring meticulous handling at the distal transition where the intima becomes delicate. Small plaque fragments are removed, and the endarterectomized lumen is irrigated with heparinized saline.

Intimal fixation sutures are placed to secure the distal endpoint. Patch angioplasty is performed using Dacron® (DuPont, Wilmington, DE, United States), polytetrafluoroethylene (PTFE), bovine pericardium, or autologous vein (most commonly the great saphenous vein), closed with a continuous 6-0 polypropylene suture.

Cossman et al. highlight the role of intraoperative stump pressure measurement in determining the need for an intraluminal shunt, traditionally obtained by inserting a 21-gauge needle into the internal carotid artery proximal to the diseased segment [34]. Although several monitoring and shunting strategies are described, available evidence suggests that overall outcomes are comparable, and selective shunting remains favored over routine avoidance [33-36]. However, this practice is considered controversial by some authors, as modern preoperative imaging, such as CT angiography and MR angiography, often provides sufficient anatomical and functional information, and additional intraoperative assessments may prolong the procedure without clear benefit.

The benefit of performing CEA within 48 hours of symptom onset remains uncertain. For symptomatic stenosis of 70-99%, CEA offers an approximate 6% mortality reduction, with neurological status assessed using the modified Rankin Scale (mRS) or National Institutes of Health Stroke Scale (NIHSS) [35,36].

Factors associated with higher perioperative risk include anticoagulation, chronic cerebral ischemia, a high carotid bifurcation, and hypertension. Potential complications include cervical hematoma, cranial nerve injury (hypoglossal, vagus, superior laryngeal, and marginal mandibular branches), arterial dissection, fistula formation, thrombosis, and, more rarely, hyperperfusion syndrome, which may result in neurological deterioration [35-39].

Perioperative Management in Carotid Revascularization

Antiplatelet therapy plays a central role in the perioperative care of carotid endarterectomy. Preoperative aspirin use is consistently associated with a lower combined risk of stroke and death, whereas dual antiplatelet therapy, despite reducing mortality in some analyses, carries a higher likelihood of perioperative bleeding and the need for reintervention. In asymptomatic patients undergoing carotid endarterectomy, low-dose aspirin (75-325 mg daily) is preferred.

These findings remain debated because other studies have not confirmed an increased bleeding risk with dual therapy. Beta-blockers should be used cautiously, as they may cause hemodynamic instability in patients with low baseline heart rates. Initiation of beta-blocker therapy is discouraged in the perioperative setting of carotid endarterectomy, whereas chronic therapy should generally be continued. In symptomatic carotid stenosis patients who do not achieve lipid targets on maximally tolerated statin therapy, ezetimibe 10 mg daily is recommended. Statin therapy prior to CEA or CAS has demonstrated meaningful reductions in 30-day adverse events, including stroke, myocardial infarction, and perioperative mortality, supporting its routine use in the preoperative optimization of vascular patients. Therapeutic anticoagulation is recommended in patients with imaging evidence of a free-floating carotid thrombus [28-30,33,35-39].

Medical Management After Carotid Revascularization

Postoperative outcomes after CEA or CAS are strongly influenced by adherence to antiplatelet therapy. Although dual antiplatelet therapy improves adherence patterns, it significantly increases the incidence of extracranial hemorrhage, particularly during the first month following CAS. Early discontinuation of P2Y12 inhibitors (<180 days) is associated with a higher risk of stroke and a greater frequency of hemorrhagic complications. For patients unable to tolerate aspirin, P2Y12-inhibitor monotherapy is an appropriate alternative. In symptomatic carotid stenosis patients not undergoing revascularization after a transient ischemic attack (TIA) or minor stroke, optimized best medical therapy, including short-term dual antiplatelet therapy followed by single antiplatelet therapy, is recommended. The combination of aspirin and ticagrelor appears beneficial when protamine is administered, but is linked to higher bleeding and ischemic event rates when protamine is omitted. Short-duration dual therapy (one to three months) followed by aspirin alone remains an effective strategy for long-term stroke prevention. Beta-blockers contribute to postoperative blood-pressure stabilization after CEA, while long-term statin therapy reduces cardiovascular complications and provides sustained cerebrovascular protection [29-31,33].

Endovascular Intervention for High-Risk Surgical Patients

Carotid artery stenting (CAS) is used as an option for patients considered high-risk for surgery. The SAPPHIRE (Stenting and Angioplasty with Protection in Patients at High Risk for Endarterectomy) trial evaluated 334 individuals and classified asymptomatic patients as high-risk when severe (70-99%) stenosis coexisted with major comorbidities, such as significant cardiac or pulmonary disease, contralateral carotid occlusion, prior cervical irradiation or surgery, recurrent stenosis, or age above 80 years [8,30,31,33,34,38].

In the SAPPHIRE trial, the three-year analysis of the pre-specified major secondary endpoint, which included death, stroke, or myocardial infarction at 30 days, along with ipsilateral stroke or death through one and three years, showed no significant difference between protected carotid stenting and endarterectomy in high-risk patients [38]. Cumulative event rates were 24.6% for stenting and 26.9% for endarterectomy, an absolute difference of -2.3% (P=0.71). Most late adverse events between years one and three were driven by non-neurologic mortality, with 19 additional deaths in the stent group and 14 in the surgery group, corresponding to an annual mortality of 7-8%. Stroke incidence was identical in both arms (15 events each; cumulative 9%), although late ipsilateral strokes occurred more frequently after stenting (four versus one event). Target-vessel revascularization remained infrequent in both groups. These findings must be interpreted with caution due to key limitations of the trial, including its small and highly selected high-risk population, incomplete three-year follow-up, lack of a contemporary medical-therapy control arm, and restricted applicability to the specific stents and embolic protection devices used. Conducted across 29 centers, the study provides useful insight for high-risk patients but offers limited generalizability to broader or lower-risk populations and to modern endovascular technologies [38].

Transcarotid Artery Revascularization (TCAR)

Transcarotid artery revascularization (TCAR) is performed through a small cervical incision that provides direct access to the proximal common carotid artery (CCA), avoiding catheter manipulation through the aortic arch. Cerebral protection is achieved by combining CCA flow interruption with temporary reversal of internal carotid artery (ICA) flow through an extracorporeal circuit [38-40].

When performed within 14 days of symptom onset, TCAR is associated with reduced procedural risk. However, outcomes are less favorable when TCAR is performed within 48 hours of the most recent symptoms, with higher rates of in-hospital stroke and stroke/death. TCAR is generally feasible when the minimal luminal diameter is ≥6 mm; smaller diameters present technical challenges and increase complication risk. Extensive calcification or atherosclerosis at the CCA access site may also preclude the procedure. When TCAR is performed between three and 14 days after symptom onset, complication rates are similar to those observed when the procedure is delayed beyond 15 days [38-40].

Data from the Vascular Quality Initiative (VQI) highlight important constraints in interpreting outcomes of TCAR, largely due to the retrospective and observational nature of the registry. Although the dataset includes a substantial number of symptomatic patients, the absence of randomization introduces selection bias, as TCAR is frequently offered to individuals with anatomical or physiological characteristics that inherently confer higher procedural risk. Moreover, the primary focus of the analysis was timing rather than direct comparison with carotid endarterectomy (CEA), limiting the ability to draw conclusions about equivalence or superiority between the techniques in broader clinical populations [37-40].

VQI data show that TCAR performed within 48 hours of symptom onset is associated with substantially higher rates of in-hospital stroke or death and increased perioperative morbidity. These early procedures also result in more frequent non-home discharges compared with delayed interventions. With only one year of follow-up and inherent confounding in observational registries, the long-term safety and true risk of urgent TCAR remain uncertain [39].

When contextualized alongside evidence from high-risk stenting trials, such as SAPPHIRE, the limitations of TCAR become more apparent. The available data do not characterize outcomes in standard-risk patients, nor do they provide insight into performance relative to modern optimized medical therapy. Taken together, the increased perioperative vulnerability during urgent intervention, the constraints of retrospective methodology, and the lack of long-term randomized evidence reinforce that CEA remains the most reliable and durable revascularization strategy in routine clinical practice, while TCAR should be reserved for carefully selected high-risk individuals until stronger comparative data emerge [40].

Discussion

This review synthesizes contemporary evidence on carotid revascularization, highlighting how heterogeneity in study design, patient selection, and outcome reporting over several decades continues to influence interpretation of comparative effectiveness and long-term durability across treatment modalities.

The lack of robust randomized trials is particularly evident for transcarotid artery revascularization. Most existing results stem from observational registries with short to intermediate follow-up, making it difficult to establish the durability or definitive comparative advantage of this technique. Early experiences suggest procedural safety in selected high-risk individuals, but current evidence is insufficient to elevate TCAR to a first-line option.

Carotid artery stenting shows variable outcomes, with low complication rates achievable in specialized centers but real-world results remaining highly dependent on anatomy, operator experience, and institutional expertise. Periprocedural stroke risk persists, particularly in elderly patients and those with complex plaque morphology, underscoring the need for careful patient selection.

In contrast, carotid endarterectomy demonstrates durable long-term outcomes with low perioperative morbidity, effective stroke prevention, and favorable cost-effectiveness, particularly in low-risk patients, supporting its role as the reference standard for most individuals with significant carotid stenosis.

Recent CREST-2 data have refocused attention on asymptomatic carotid disease. The trial was designed on the premise that carotid endarterectomy and stenting are complementary rather than competing strategies, incorporating rigorous operator credentialing and procedural oversight to optimize patient safety and fairly assess revascularization in the era of optimized medical therapy.

Carotid artery stenting can be an effective and safe alternative to carotid endarterectomy when strict anatomical and clinical selection criteria are applied, as exemplified by the CREST-2 protocol. Exclusion of patients with unfavorable features, such as complex aortic arch anatomy, severe vessel tortuosity, extensive calcification, proximal or distal obstructive disease, or contrast intolerance, contributes to optimized outcomes, particularly in experienced centers. However, these rigorous anatomical and clinical selection criteria result in highly selected populations, limiting generalizability and highlighting the inherent selection bias of contemporary trials. Consequently, the favorable results observed for CAS should be interpreted with caution and may not be extrapolated to broader, unselected patient populations, especially elderly individuals or those with complex anatomy [33-39].

Evidence in asymptomatic carotid stenosis originates from a therapeutic era that differs from current practice. Improvements in best medical therapy have markedly reduced ipsilateral stroke risk, resulting in a low absolute risk in asymptomatic disease. Therefore, carotid intervention for the prevention of cognitive impairment in patients with 70-99% asymptomatic stenosis is not currently recommended, and further evidence is needed to establish a causal link with cognitive decline.

Collectively, the available evidence reinforces the need for a patient-specific approach to carotid revascularization. Carotid endarterectomy remains the most dependable strategy for individuals at low operative risk, supported by decades of consistent long-term outcomes. Carotid artery stenting maintains a defined role in patients with anatomical or clinical features that render surgery less favorable, while transcarotid artery revascularization represents a promising but still incompletely validated option. Robust randomized trials-particularly those evaluating TCAR and long-term comparative durability-are critically needed to strengthen the evidence base and guide future clinical decision-making [35-40].

Limitations

This study has several important limitations. The evidence base relied largely on observational and non-blinded studies, which limits control over confounding factors. Although this work was conducted as a structured review, employing the PICO framework and a PRISMA-guided selection process to reduce bias, restricting the search to open-access articles may have narrowed the scope of available evidence. The limited number of high-quality randomized clinical trials further weakens the strength of comparisons across treatment modalities. The use of a random-effects model may introduce statistical imprecision in the presence of substantial heterogeneity; however, it was chosen to support broader generalizability of findings, unlike fixed-effects models, which are unsuitable for cross-study comparisons. Finally, potential publication bias and variability in outcome reporting over several decades may have influenced the overall interpretation of the results.

## Conclusions

In our study, carotid endarterectomy (CEA) was associated with lower rates of perioperative stroke and the composite outcome of death or stroke, whereas carotid artery stenting (CAS) was associated with a lower incidence of perioperative myocardial infarction. These findings reflect a recognized procedural trade-off rather than clear overall superiority. Importantly, when performed in experienced centers with appropriate patient selection, long-term outcomes of CEA and CAS appear broadly comparable, underscoring the need for individualized treatment decisions based on clinical risk profile and anatomy.
